# Serbian Traditional Goat Cheese: Physico-Chemical, Sensory, Hygienic and Safety Characteristics

**DOI:** 10.3390/microorganisms10010090

**Published:** 2021-12-31

**Authors:** Katarina G. Mladenović, Mirjana Ž. Grujović, Sunčica D. Kocić-Tanackov, Sandra Bulut, Mirela Iličić, Jovana Degenek, Teresa Semedo-Lemsaddek

**Affiliations:** 1 Department of Science, Institute for Information Technologies, University of Kragujevac, Jovana Cvijica bb, 34000 Kragujevac, Serbia; katarinam@kg.ac.rs; 2 Faculty of Technology, University in Novi Sad, Cara Lazara 1, 21000 Novi Sad, Serbia; suncicat@uns.ac.rs (S.D.K.-T.); sandra.bulut@gmail.com (S.B.); panim@uns.ac.rs (M.I.); degenek.9.19.d@uns.ac.rs (J.D.); 3 CIISA—Centro de Investigação Interdisciplinar em Sanidade Animal, Faculdade de Medicina Veterinária, Universidade de Lisboa, Avenida da Universidade Técnica, 1300-477 Lisboa, Portugal; tlemsaddek@fmv.ulisboa.pt

**Keywords:** traditional goat cheese, ripening, physico-chemical characteristics, sensory properties, antibiotic resistance, safety

## Abstract

This research project aimed to investigate the physico-chemical, sensory, hygienic and safety characteristics of raw goat milk, whey, brine and traditional goat cheese during the ripening period of 28 days. Physico-chemical parameters included the determination of dry matter, fat, ash, protein, pH, water activity and NaCl content. The presence of Enterobacteriaceae and fungi was estimated on milk and cheese samples, and a sensory panel evaluated the products’ features and acceptability during ripening. The results show that the cheese under study belongs to the acid full-fat cheese group. A consumer panel attributed high scores to the goat cheese, until the 21st day of ripening. After this period, the overall features altered significantly, including augmented bitterness, odor intensification and the development of molds on the surface. The presence of fungi, associated with Enterobacteriaceae, suggests that the hygiene of the production processes needs to be improved. Regarding microbial safety, the detection of putative pathogens and antibiotic resistances recommend an active surveillance of traditional foods to avoid foodborne infections and/or the dissemination of resistant microorganisms along the food chain.

## 1. Introduction

Fermented foods are a very important part of the human diet, and fermentation has been used to improve the shelf life, safety, digestibility and nutritional value of diverse foods [1]. Worldwide there are many geographical areas known for their traditional way of producing dairy products. Most of these fermented foods are produced using various manufacturing techniques and raw milk [2], without the addition of commercial starter cultures [3,4]. Hence, the quality of the raw materials is essential to assure the quality of the final product. Regarding Serbian goat cheese, quality relies on the use of milk collected from animals reared in organic farming systems. This organic milk contains more dry matter and nutrients; hence, its usage leads to the obtention of dairy products with exceptional nutritional and functional properties [5]. Goat’s milk harbors a specific taste, and it is known to cause less allergic reactions than cow’s milk. The absence of adverse reactions is due to its low or minimal level of the αs1-casein fraction [6]. Goat’s milk is generally more easily digested (the fat globules are smaller) and represents a good source of calcium, phosphorus and vitamins [5,6].

Members of the Enterobacteriaceae family are an important members of the microbial communities present in raw milk and cheeses (such as Montasio and Urfa cheeses) [7,8,9]. Their presence is usually attributed to hygiene problems during milking and manipulation. The antibiotic resistance profile and detection of degradative enzymes by Enterobacteriaceae isolated from raw goat’s milk were determined by Ramos and Nascimento [8]. Additionally, molds that can be found in cheeses may originate from raw materials such as milk or may be added on purpose during cheese production, as starter cultures, or they may be present due to cross-contamination from the environment [10].

Antimicrobial drug resistance (AMDR) is one of the top 10 global public health threats facing human and animal health in the 21st century, due to the lack of therapeutic options [11]. In addition, the human population may be exposed indirectly to antibiotic-resistant bacteria and antibiotic-resistant genes (ARGs) via contact with or consumption of contaminated food products (e.g., meat, eggs, milk and dairy products) [12]. Numerous reports have described the presence of large quantities of antibiotic-resistant bacteria and ARGs in various food products (ready-to-eat meat, cooked meat and bulk milk) from various animal sources, such as cattle, poultry, swine, goat and sheep, and from different stages of food production [13,14]. In addition, milk is considered an excellent culture medium for gene transfer by conjugation and has been reported to have an efficiency that is 10 times higher than that of laboratory culture medium [15]. Some studies have described the occurrence of multidrug resistance (MDR) in members of the Enterobacteriaceae family isolated from dairy products [16,17]. Therefore, there is a need for investigation on the dissemination of microbial pathogens and AMDR through the food chain.

In this context, the present study evaluated the physico-chemical properties of Serbian goat milk, cheese, brine and whey as well as the presence of Enterobacteriaceae and molds. Moreover, the sensory features of the traditional goat cheese were also assessed during 28 days of ripening. To further elucidate the safety of the foods under study, enterobacteria were evaluated regarding antibiotic resistance profiles.

## 2. Materials and Methods

### 2.1. Cheese Production and Sampling

The cheese under study was manufactured in a countryside household in the Pajsijević village (Central Serbia) in spring of 2021. Fresh hand-milked goat milk (pH 6.6) was collected after morning and evening milking in an enameled pot and stored at 10–12 °C (temperature in cellar). The raw milk was filtered through gauze into an enameled pot and heated to 32 °C, and a liquid rennet Sirela (MTM Sirela, Čačak, Serbia) (85% chymosin and 15% pepsin) was added in the amount of 28 mL for 10 L of milk, with constant stirring. After 30–60 min, the whey separated, and the clotted mass was cut into cubes using a knife. The cheese was mixed with a wooden spoon by turning it over (what was at the bottom of the sieve was moved to the top, and vice versa) and covered with a gauze. After a rest time of 15 min, the entire contents were transferred to a new, large gauze and left to rest overnight, to enable whey removal. The next morning, the cheese was salted (10 g of salt per layer of curd) and cut into cubes. The cubes were placed in wooden boards and poured with brine (whey that is salted (1 L of whey was salted with 50 g of salt)). A cotton cloth was placed on top, which was then covered with a wooden board and finally with a 1 kg marble stone and stored in a cellar (ripening period) at 15–16 °C, for 28 days. Ten liters of milk were needed to produce 2.65 kg of cheese. Generally, the cheese is consumed fresh, i.e., immediately after production. This study investigated the maximum shelf life of cheese during ripening in described conditions in which the cheese could be safely consumed.

For the present study, cheese samples (200 g) were collected from the container immediately after manufacturing, i.e., immediately before storage (day 0) and during the next 28 days, i.e., at the 7th, 14th, 21st and 28th day of ripening; aseptically transported to the microbiology laboratories at the Department of Science, Institute for Information Technologies, University of Kragujevac and at the Faculty of Technology, University of Novi Sad; and kept at 4 °C until processing. The analysis occurred in the next 24 h after sampling. Goat’s milk, brine and whey were also sampled.

### 2.2. Physico-Chemical Analysis

Chemical analysis of goat cheese was performed by using standard methods: dry matter content after drying cheese samples at the temperature of 105 ± 1 °C [18]; milk fat content according to Van Gulik’s acidobutyrometric method of fat in cheese, by using a special cheese butyrometer [19]; ash content by annealing the sample at 550 °C [20]; total protein content according to *Kjedahl* [21]; pH value by measuring with an electric pH meter (pH Spear, Eutech Instruments, Oakton, England); titratable acidity according to Soxhlet–Henkel [22]; water activity (*aw* value) by using LabSwift-aw device (Novasina AG, Lachen, Switzerland); NaCl content was determined according to Carić et al. [23], and fat in dry matter calculated with the formula: fat (%) × 100/dry matter (%) [24]. Milk and whey fat contents were assessed according to Gerber [25]. The pH value, water activity (aw value) and NaCl content of brine were investigated as previously explained. All determinations were performed in triplicate.

Milk and cheese color were evaluated using the photoelectric tristimulus colorimeter (CHROMAMETER CR-400, Konica Minolta) with a CR-A33F special extension for the determination of cheese color parameters. The working principle was based on the measurement of reflected color and differences in color values on different cheese surfaces, including whiteness (L*), red/greenness (a*), and yellow/blueness (b*), and dominant wavelength (λ). All assays were performed in triplicate. The total color difference (ΔE) was calculated between measurements of cheese sample after production and cheese samples during ripening according to ΔE = {(L_0_*−L*)^2^ + (a_0_*−a*)^2^ + (b_0_*−b*)^2^}^1/2^, where L_0_*, a_0_* and b_0_* are the value of cheese color components after production, and L*, a* and b*are the value of cheese color components during ripening [26].

### 2.3. Sensory Evaluation

Sensory evaluation of goat cheese was performed according to a standard method [27]. Goat cheese samples were stored at 4 °C until the moment of sensory evaluation. Before the sensory evaluation, a panel of 8 evaluators (six females and two males) between 28 and 50 years old were trained in order to familiarize them with the product. During the evaluation, the test samples had a temperature of 14 °C ± 2 °C. The cheese sample (200 g) was divided into eight equal proportions and presented simultaneously to each of the eight consumers. Evaluators assessed each particular element of quality such as appearance, color, texture, cross-section, odor and taste. The quantitative descriptive method and the five-point system, a scoring range from 1 to 5, (from dislike extremely (1) to like extremely (5)), were used for sensory evaluation [28]. Cheese sensory analysis was performed on days 0, 7, 14, 21 and 28. Sensory properties are presented graphically in the 2-D column as mean values.

### 2.4. Microbiological Analysis

#### 2.4.1. Enumeration, Isolation and Identification of Enterobacteriaceae

Samples were collected from the center of the goat cheese container, using a sterile spoon, on days 0, 7, 14, 21 and 28. The working section (10 g) was added to 90 mL of 2% (*w*/*v*) sodium citrate, previously sterilized and heated at 45 °C, and thoroughly mixed in the vortex until complete homogenization. Subsequently, successive ten-fold dilutions in 2% (*w*/*v*) sodium citrate were prepared and inoculated on selective media for Enterobacteriaceae counts, specifically Violet Red Bile Agar (VRBG, Oxoid, Hants, UK) [29,30,31]. In addition, 1 mL of pure goat’s milk was also incorporated into the same medium. Characteristic colonies (purple/pink, with or without halos) were transferred to Tryptone Soya Agar (TSA, Torlak, Belgrade, Serbia) for further characterization that is well described by Jesumirhewe et al. [32]. Briefly, Gram-negative, catalase-positive and oxidase-negative isolates were submitted to biochemical tests (production of indole, ONPG, urease, H_2_S, TDA and VP assays) and growth on selective media, such as citrate agar (Torlak, Belgrade, Serbia) and HiChrome coliform agar (Sigma-Aldrich Chemie GmbH, Buchs, Switzerland). Presumptive Enterobacteriaceae were further analyzed using Microgen GN-A and Microgen GN-B test (Microgen, Germany), and results were interpreted according to Microgen ID (software version 2.0.8.33). Lastly, identification was confirmed using matrix-assisted laser desorption/ionization time-of-flight mass spectrometry (MALDI-TOF MS) using the corresponding values to match manufacturer suggested results ≥ 2.00 [33]. Bacterial isolates of interest were kept in a 20% glycerol/medium mixture at −80 °C at the Faculty of Science, University of Kragujevac.

The total number of aerobic mesophilic bacteria was also enumerated in goat milk and cheese samples according to the standard method [34].

#### 2.4.2. Screening for Proteolytic and Lipolytic Activities

The proteolytic activity was examined according to Harrigan and McCance [35], with slight modifications: The substrate was formed by mixing nutrient agar medium and milk (1.6% fat) in a proportion of 1:1. After bacterial inoculation, the plates were incubated 24 h at 37 °C. The appearance of a clear zone around the colonies was considered as proteolytic activity. Regarding lipolytic activity, this trait was evaluated according to Harrigan and McCance [35], with minor adjustments: The substrate was prepared by adding 4% egg yolk to nutrient agar, after which it was inoculated and incubated for 24 h at 37 °C. Appearance of an opalescent zone around the bacterial colonies confirmed their lipolytic activity. *Bacillus subtilis* ATCC 6633 was used as a positive control, while *E. coli* ATCC 25922 was used as a negative control.

#### 2.4.3. Antibiotic Resistance Profiles

To determine antibiotic resistance profiles, the disk diffusion method was applied following the methodology described by European Committee on Antimicrobial Susceptibility Testing (EUCAST). Four antibiotics, representing distinct classes and modes of action, were selected (Gentamicin 10 µg, Amoxicillin 25 µg, Chloramphenicol 30 µg, Tetracycline 30 µg (Bioanalyse, Ankara, Turkey). According to EUCAST breakpoints [36] zones of inhibition (in mm) were determined.

#### 2.4.4. *E. coli* O157 Rapid Latex Agglutination Test

*E. coli* O157 rapid latex agglutination test (Microgen, Germany) is designed to confirm the identity of *E. coli* serotype O157 isolated from samples of human origin or foods [37]. The methodology enables rapid differentiation among *E. coli* serotypes. Latex particles are coated with antibodies raised against the somatic O157 lipopolysaccharide antigen of *E. coli* O157. When sensitized latex particles are mixed with an *E. coli* suspension containing the antigens O157, a sensitive and specific immunochemical reaction takes place, causing the finely dispersed latex particles to agglutinate into aggregates that are easily visible to the naked eye.

#### 2.4.5. Enumeration, Isolation and Identification of Molds

The total number of molds present in milk, whey, brine and cheese was evaluated according to standard procedures [38]: 20 g of cheese or 20 mL of milk, brine and whey were homogenized with 180 mL of Buffered Peptone Water (Merck, Darmstadt) by shaking for 15 min, at 200 rpm (Unimax 1010, Heidolph, Schwabach, Germany). Successive ten-fold dilutions were prepared, inoculated on selective media Dichloran Rose Bengal Chloramphenicol (DRBC) Agar (Merck, Darmstadt, Germany) and incubated for 7 days at 25 °C ± 2 °C. The colonies were counted, and results are expressed as mean values in CFU/g or CFU/mL. In order to obtain pure cultures and perform identification based on macromorphological properties mold colonies suspected to belong to the *Acremonium*, *Alternaria* and *Cladosporium*, *Geotrichum* genera were subcultured on Sabouraud Maltose Agar (SMA) (Merck, Darmstadt), while presumptive *Aspergillus*, *Penicillium* and *Talaromyces* genera were subcultured on Czapek Yeast Autolysate Agar (Merck, Darmstadt) and incubated for 7 days at 25 °C ± 2 °C. The identification of molds was performed based on macroscopic (diameter, color, texture, pigmentation and reverse of the colony) and microscopic (metules, phialides, conidia, hyphaes, etc., and their length, diameter, size and shape) characteristics, according to criteria described by Samson and Frisvad [39] and Pitt and Hocking [40].

### 2.5. Data Analysis

Results obtained are presented as mean ± standard deviation, and the *t*-test (Statistica 9.0) was applied to determine the significance of the differences observed.

## 3. Results

### 3.1. Physico-Chemical Analysis

The results observed for goat’s milk, whey and cheese during 28 days of ripening are displayed in Table 1 and Table 2. The dry matter in milk was 14.21%, and 7.7% in whey, while in cheese the highest content of dry matter was detected on the 21st day (52.64%), and the lowest content was measured on day 0 (38.91%). Total protein content in milk was 3.58%, while in whey it was 1.36% (Table 1). In cheese (Table 2), the highest values for protein content were also observed on the 21st day (15.31%) and the lowest on day 0 (12.31%). The pH values for milk and whey were 6.52, whilst the cheese pH ranged between 4.75 and 6.55. Salt amount varied from 0.69% to 0.96%. Total ash content ranged between 2.03% and 1.30%, with the lower values corresponding to longer ripening periods.

The goat cheese colors were measured using the CIE Lab color system. It was evident that goat cheese samples (Table 2) presented higher values (L*, a, b) in comparison to milk (Table 1). The whiteness of goat cheese did not change significantly during 21 days of ripening. However, the storage period of time had a significant effect on red/greenness (a) and yellow/blueness (b) coefficients. The Y value of goat cheese changed from 73.09.15% (day 0) to 70.95% (day 21), while dominant wavelengths during storage ranged from 573.24 nm (day 0) to 574.27 nm (7th day). Total color differences of goat cheese (ΔE) were estimated, and the highest change was observed after 14 days of ripening (2.40), with the lowest alterations occurring after 7 days (1.04).

### 3.2. Sensory Properties

**Figure 1:**
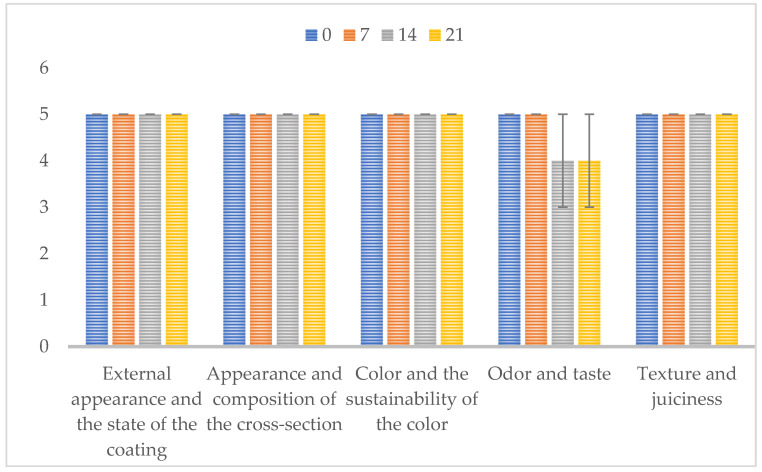
Sensory properties of traditional goat cheese during ripening.

The goat cheese sensory features were analyzed during 28 days of ripening, using the 5-point hedonic scale, based on the evaluation of appearance, color, texture, cross-section, odor and taste. The results obtained are shown in Figure 1. On day 0, consumers described the cheese as compact, with a cross-section without cavities, having a typical odor and mild taste, as is usually associated with this food product. After 7 days of ripening, the cheese was defined as harboring an acceptable color, typical odor and a slight bitter taste. At day 14, they indicated an intense taste with increased bitterness, while after 21 days of ripening the cross section presented a small number of cavities, characteristic smell and pronounced bitterness. On the 28th day it was not possible to carry out the predicted sensory analysis, due to the development of molds on the cheese surface. Overall, the panel members reported excellent sensory characteristics during the first three weeks of ripening, after which the cheese started to present a bitter taste and a more intense odor, which culminated in the decrease in attributed points.

### 3.3. Microbiological Analysis

#### 3.3.1. Enumeration, Isolation and Identification of Enterobacteriaceae

The counts of total enterobacteria present in goat’s milk and cheese samples were assessed using the VRBG medium (Table 3). Briefly, microbial loads ranged between 9.09 × 10^4^ CFU/g (day 0) and 1.24 × 10^8^ CFU/g (14th day) of cheese, whilst in milk the levels were similar to those observed at cheese day 0. As aforementioned, after 28 days of ripening molds developed on the surface of the cheese, which prevented the isolation of enterobacteria for further analysis.

The total number of aerobic mesophilic bacteria present in goat’s milk and cheese samples were addressed using a nutrient agar (Table 3). Microbial loads ranged between 3.6 × 10^6^ CFU/g (day 0) and 5.24 × 10^11^ CFU/g (14th day) of cheese. It could be noticed that the initial number of aerobic mesophylic bacteria was lower compared to the total number of enterobacteria in milk. However, in cheese samples, the number of aerobic mesophylic bacteria was higher compared to the total number of enterobacteria.

**Figure 2:**
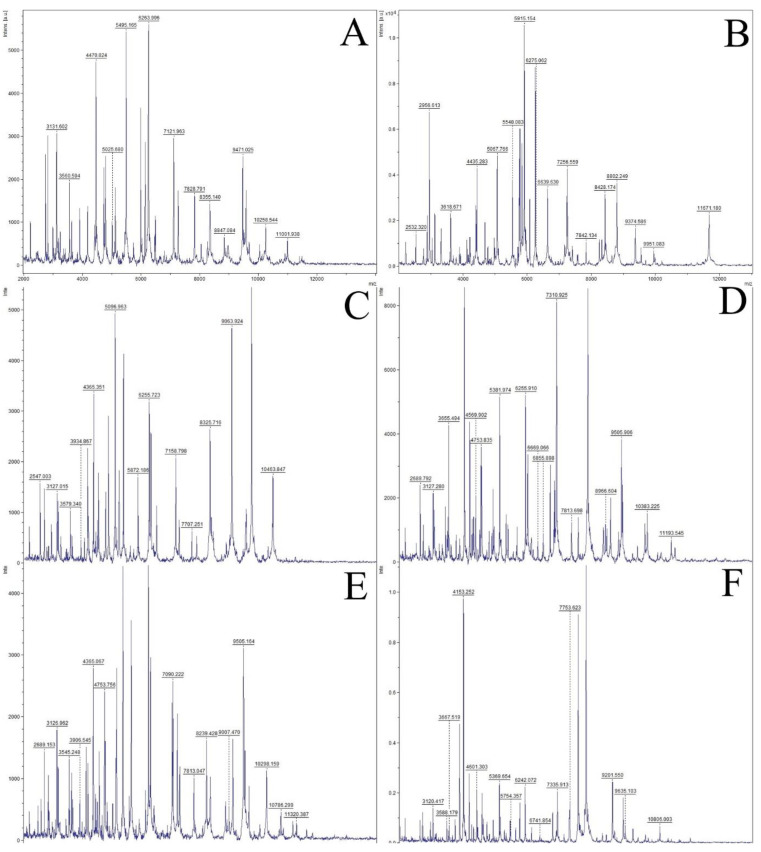
Mass spectra of the isolated bacteria (**A**)—*P. mirabilis*; (**B**)—*Pseudomonas* spp.; (**C**)—*E. coli;* (**D**)—*E. cloacae*; (**E**)—*E. asburiae*; (**F**)—*R. aquatilis*).

**Figure 3:**
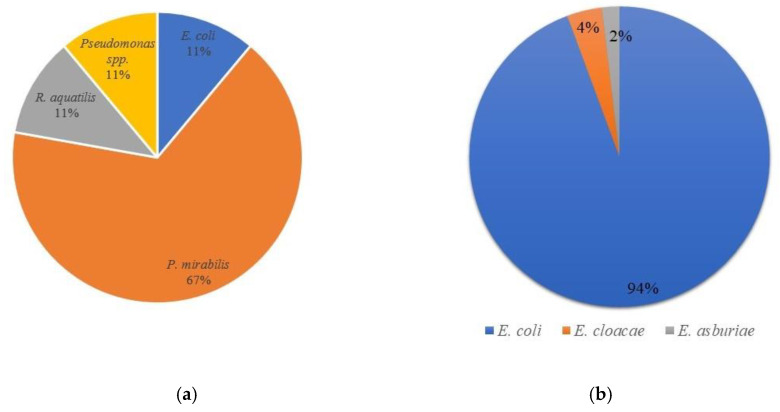
Distribution of Enterobacteriaceae in milk (**a**) and cheese (**b**).

After plate counting on VRBG agar, presumptive enterobacteria were isolated (10 bacteria per plate) and submitted to conventional microbiological characterization. All Gram-negative, catalase-positive and oxidase-negative bacteria were further analyzed by biochemical tests and with Microgen GN-A and GN-B tests (Table 4). All isolates were incubated in HiChrome coliform agar and citrate medium, where a characteristic growth (color of the colonies) and color of the medium were shown, respectively. For additional confirmation of species allocation, MALDI-TOF mass spectrophotometry was applied (Figure 2). The results obtained identified the following microorganisms: *Escherichia coli* (54 isolates), *Proteus mirabilis* (6 isolates), *Rahnella aquatilis* (1 isolate), *Pseudomonas* sp. (1 isolate), *Enterobacter cloacae* (2 isolates) and *Enterobacter asburiae* (1 isolate). It was observed that *E. coli* is the most dominant species in cheese, while *P. mirabilis* is the dominant in goat milk (Figure 3).

#### 3.3.2. Proteolytic and Lipolytic Activities

The results of the investigation of the proteolytic and lipolytic activity of isolated bacteria from goat’s milk and cheese indicated that none of the isolates possess proteolytic or lipolytic activities (Table 3). The exceptions were *P. mirabilis* GM-6 and *R. aquatilis* GM-9, both isolated from milk, which showed proteolytic activity.

#### 3.3.3. Antibiotic Resistance Profiles

Goat’s milk and cheese isolates showed different sensitivity to the tested antibiotics (Table 3). All isolates from the genus *Escherichia* showed resistance to gentamicin, but they were sensitive to amoxicillin, chloramphenicol and tetracycline. *E. asburiae* and *R. aquatilis* isolates were sensitive to all tested antibiotics, while *E. cloacae* isolates showed resistance to amoxicillin and tetracycline. Isolates from the genus *Proteus* showed sensitivity to gentamicin and chloramphenicol, but they were resistant to amoxicillin and tetracycline. *Pseudomonas* sp. showed resistance to tetracycline and amoxicillin. In general, there was no isolate that showed full resistance to antibiotics.

#### 3.3.4. *E. coli* O157 Rapid Latex Agglutination Test

According to the *E. coli* O157 rapid latex agglutination test, none of the tested *E. coli* isolates belong to the serotype *E. coli* O157.

#### 3.3.5. Isolation and Identification of Molds

The enumeration of molds, as well as their isolation and identification, was determined in milk, whey, brine and cheese samples. In milk, 20 CFU/mL were detected, including *Alternaria alternata* and *Geotrichum candidum*. In whey, the total number of molds was 100 CFU/mL, while the identified species corresponded to *Acremonium strictum*, *Penicillium brevicompactum*, *Penicillium chrysogenum*, *Penicillium expansum*, *Penicillium glabrum* and *Talaromyces albobiverticillus*. The total number of molds in brine matched 80 CFU/mL, and the identified species were *Acremonium strictum*, *Penicillium aurantiogriseum*, *Penicillium glabrum* and *Penicillium thomii*. On the first day of cheese ripening (day 0), the total number of molds was 200 CFU/g, and the following species were identified: *Cladosporium macrocarpum* and *Penicillium aurantiogriseum*. On the 7th and 14th day of ripening, the total number of molds was less than 100 CFU/g, whereas in the 21st day, the total number corresponded to 5.4 × 10^5^ CFU/g, and *Aspergillus flavus* was the dominant species (Supplementary Figures S1–S17).

## 4. Discussion

To our knowledge, this is the first report on the physico-chemical, sensory and microbiological properties of Serbian goat’s milk and traditional cheese during 28 days of ripening. This product was manufactured at a local Serbian village, using organic unpasteurized goat’s milk, in the traditional way, with enzymatic rennet and without the addition of microbial starters.

A previous study addressing the physico-chemical characteristics of organic goat’s milk observed 13.14% of dry matter, 4.29% of milk fat, 4.42% of total proteins and 0.90% of ashes [5], which is in accordance with our findings. These authors also analyzed a four-day-old goat’s cheese, which harbored 38.91% of dry mater, 14.4% of fat, 15.39% of total proteins and 2.03% of ash. Similar results were observed in the present study at day 0 or after 7 days of ripening. Moreover, considering the fat in dry matter, the tested goat cheese belongs to the group of full-fat cheeses. The quality of goat’s milk fat and protein is an important factor because it defines the ability of milk to be processed and has a relevant role in the nutritional and sensory quality of the products obtained from it [41].

The sensory characteristics and color of goat cheese are fundamental to consumer acceptance. Cheese color and flavor depend on the presence of fat globules and casein micelles. Our results show that goat cheese samples have significantly higher L*, a and b values compared to goat’s milk. Regarding goat milk and cheese samples, lightness values ranged from 82.44 (goat milk) to 88.49 (cheese samples after production. The decrease in lightness during production and the ripening of goat cheese is due to a lower pH value, which results from a change in the hydration of protein, leading to decreased moisture. Value *a* (red/greenness) of cheese samples decreased, while *b* values increased during the 21 days of ripening. These results could be related with the cheese’s yellowness caused by moisture loss. The highest changes in cheese color occurred after 14 days of ripening, as a result of the microbiological activity of non-starter lactic acid culture and complex biochemical processes (proteolysis, lipolysis, oxidation processes) [42,43].

Regarding the organoleptic features and consumers’ acceptance of the goat cheese under analysis, the results show that Serbian traditional cheese presented excellent sensory characteristics until the 3rd week of ripening, after which the smell and bitter taste started to be noticeable. This perception reflected on the points attributed by the sensory panel, which decreased from the 14th day onwards. These undesirable features could have resulted from complex biochemical transformations attributed to specific production steps (e.g., type and concentration of the enzyme used for milk coagulation) and/or to the microbial communities present [44]. Raw milk cheese ripens more quickly and develops a stronger flavor than cheese produced with pasteurized milk. Often, the increased activity of enzymes and non-starter lactic acid bacteria (NSLAB) can cause cheese bitterness. NSLAB are known to affect cheese quality and to flavor intensity [45]. As the taste of the goat cheese under study began to change intensively on the 21st day and *A. niger* appeared, no sensory evaluation was performed on the 28th day of ripening, and the color of the cheese was not assessed.

For consumers’ acceptance it is fundamental to trust the microbiological quality and overall safety of the cheeses produced under traditional domestic conditions, at the farm level. The microbial communities present in dairy foods, produced from raw milk, rely primarily on the microorganisms from the raw materials, the hygiene during production and the possibility of cross contamination during manufacture or storage [46]. In fact, the microbial composition of raw milk may have health-related implications, due to contamination with pathogens associated with severe foodborne illnesses [47]. Moreover, according to Oikonomou et al. [48], the specific composition of the milk microbiota directly affects the subsequent development of dairy foods’ characteristics. Microorganisms can bring about the fermentation of milk through the production of lactate and have a variety of different impacts on the sensory, texture, flavor and organoleptic properties of the resultant dairies.

Coliform bacteria, such as members of the Enterobacteriaceae family, are considered fundamental indicators of the hygiene practices during the production processes [49]. Specifically, regarding the manufacture and ripening of cheese, it has been shown that enterobacteria are present [50] and slowly decrease during ripening [51]. Other authors also demonstrated high levels of coliform contamination (from under 1 to 7.89 log CFU/mL) in different types of cheese, produced using traditional methods from cow’s, goat’s, sheep’s or mixed milk [3,4,49]. Similar results were verified in the present study, since the Enterobacteriaceae levels ranged from 9.09 × 10^4^ to 1.24 × 10^8^ CFU/g of cheese, from the first to the last day of ripening, respectively. The number of enterobacteria depends on the pH value of the cheese; as it becomes more acidic, the number of Enterobacteriaceae decreases [52]. In the present investigation, the highest microbial count was observed on the 14th day, when the pH marked a value of 5.3.

Bacterial identification on the species level is a very important aspect of microbiology. The VRBG agar was used for isolation of enterobacteria. Further, Gram-negative, catalase-positive and oxidase-negative isolates were submitted to biochemical tests and growth on selective media, such as citrate agar and HiChrome coliform agar (see Materials and Methods section) in order to detect presumptive enterobacteria. Isolates were further analyzed using Microgen GN-A and Microgen GN-B tests. These are the basic tests used for Enterobacteriaceae identification [32]. However, not all microorganisms are reliably identified by biochemical methods. These methods often give wrong results at an unacceptable rate [53]. In addition, many authors have stated that MALDI-TOF MS has been successfully used for the identification of a wide array of bacteria [32,53,54,55]. According to Jesumirhewe et al. [32], the difference in identification between commercial identification systems (such as Microgen or API strips) and MALDI-TOF MC exists at the level of the genus (57.2%) and at the level of the species (33.1%), which was confirmed in our study. Hence, MALDI-TOF MS can replace conventional biochemical methods because identification is much more accurate.

Enterobacteriaceae capable of synthesizing proteolytic and lipolytic enzymes are largely responsible for the deterioration of milk and dairy products, which may cause various issues in the dairy industry [56,57]. In cheese production, these enzymes destabilize casein micelles and may modify or even prevent milk coagulation, which can directly affect the formation of the intended product. Additionally, these bacteria can affect the foods’ sensory properties, such as color, odor, flavor and texture [57,58]. Such modifications can directly affect the acceptance or rejection of the product by the consumer. The bacterial synthesis of lipolytic enzymes has also been shown to be relevant in the food industry, due the direct influence of these enzymes on the sensory properties, particularly flavor and texture. Masiello et al. [56] isolated lipolytic representatives of the Enterobacteriaceae family (genera *Serratia, Enterobacter* and *Raoutella*) from pasteurized milk samples. The authors indicated that diverse bacteria found in pasteurized milk, exhibiting phenotypic characteristics such as production of lipolytic and proteolytic enzymes, can result in milk spoilage. Similar results were verified in the present study since *R. aquatilis* showed proteolytic activity. Although *Pseudomonas* spp. represents the genus of psychotropic microorganisms most related to proteolytic activity in raw milk [8], the goat cheese isolate did not show this property. The other genera of the Enterobacteriaceae family, such as *Enterobacter* and *Klebsiella*, usually do not display this trait [59], which was confirmed by our results. None of the tested bacteria showed lipolytic activity.

The presence of Gram-negative bacteria in dairy foods is common [3,4,47,60,61]. Although these microorganisms are frequently considered indicators of poor hygiene and may constitute a health risk if pathogenic and or antibiotic resistant species are present, some may also play important roles in dairy fermentations by contributing to the sensory quality of the dairy products [52,62]. Enterobacteriaceae present in the milk and cheese probably come from the udder of the animal, hands of the manipulators, or the utensils or equipment used [41,63,64]. Level variations along time can be associated with differences in feed. Callon et al. [65] suggested that other factors, such as weather conditions and the animal health, were also important. The presence of Enterobacteriaceae has been detected in many varieties of goat cheese [46,50] and, in the present research, 53 Enterobacteriaceae were isolated from cheese and 9 from milk. *Proteus mirabilis* was predominant in milk, while *E. coli* was the most frequently recovered species from cheese. After the agglutination test, *E. coli* isolates were confirmed as not belonging to O157 serotype, which can be interpreted as a relevant result, as it points towards the microbial safety of the final product.

Antibiotics have been extensively used in human and veterinary medicine, and in agricultural settings, for the treatment of infections, growth enhancement and prophylaxis, potentially leading to the selection of drug- and multidrug-resistant bacteria [12,66,67]. In fact, the association between the uncontrolled use of antibiotics in human and veterinary medicine and the selection of multidrug-resistant bacteria in humans and cattle has been previously reported [66]. Even though antimicrobial-resistant determinants may be associated with non-pathogenic bacteria, horizontal gene transfer (HGT) events can lead to their transference to pathogenic microorganisms, such as several members of the Enterobacteriaceae family, increasing the prevalence and dissemination of antimicrobial resistance along the food chain [12,68,69]. The likelihood of HGT is even greater if resistance genes are located in mobile genetic elements [70]. Consequently, enteric bacteria such as *E. coli*, *Enterococcus faecalis* and *Salmonella* spp. are not only resistant to multiple antibiotics given to animals but also to antibiotics made available to humans [71]. These bacteria are natural inhabitants of the intestinal tract of humans and cattle, providing a potential reservoir for the contamination of distinct settings [66]. Regarding dairy species, the use of antibiotics is usually associated with the treatment of mastitis [72]. Hence, food products of animal origin may harbor antimicrobial resistant bacteria, constituting a potential health risk for consumers [73]. Additionally, studies have shown that *E. cloacae* strains are often isolated from dairy products. However, studies of the virulence factors of these strains and their MDR potential are still scarce. Nonetheless, in our research low levels of resistance were observed. All isolates showed susceptibility to chloramphenicol. *E. coli* isolates were resistant to gentamicin and susceptible to other tested antibiotics. *E. cloacae*, *P. mirabilis* and *Pseudomonas* spp. isolates showed resistance to amoxicillin and tetracycline, while *E. asburiae* and *R. aquatilis* isolates were susceptible to all antibiotics.

Mold contamination in cheese can affect its organoleptic properties, influence the cheese quality and potentially produce secondary toxic metabolite mycotoxins, and therefore poses a potential health risk to consumers. Mycotoxin cheese contamination can occur indirectly via milk contamination or directly through mycotoxin producing spoilage molds [74]. However, mold growth on the cheese surface does not necessarily indicate the presence of mycotoxins in cheese. Opinions of various authors on the question of cheese as a substrate for the development of mycotoxins are divided, and the general opinion is that cheese is a better medium for mold growth than mycotoxin production [10,75], especially if cheese is correctly stored at a low temperature (5 to 7 °C) [10].

The presence of fungi in raw milk can be influenced by the physiological state of the animal, as well as the weather, feeding and season [63,65]. Fungus can play a major role in dairy fermentation due to its physiological and biochemical characteristics, including the ability to utilize lactose or galactose, high proteolytic or lipolytic activities and the ability to grow at low temperatures and tolerate high salt concentrations [76]. The genera most frequently associated with raw milk include *Penicillium*, *Geotrichum*, *Aspergillus*, *Mucor* and *Fusarium* [77]. At the species level, *Fusarium merismoides, Penicillium glabrum, Penicillium roqueforti, Aspergillus fumigatus, Engyodontium album*, as well as species of the *Cladosporium* and *Torrubiella* genera, are common [78]. In the present research, brine and whey were characterized with dominant *Penicillium species*, while *Alterinaria alterinata, Geotrichum candidum* and *Cladosporium macrocarpum* were less dominant. The presence of the aflatoxigenic species *Aspergillus flavus* was observed on the cheese on the 21st day of ripening. According to the obtained results the molds found in cheese samples may indicate poor hygienic conditions during cheese production and/or storage, such as *Cladosporium macrocarpum*, or from the brine, such as *Penicillium aurantiogriseum*. Since the toxigenic mold *Aspergillus flavus* was found only in the cheese sample, after the 21st day of ripening, it is most likely that its occurrence resulted from cross-contamination.

## 5. Conclusions

The present investigation evaluated the physico-chemical, sensory, hygienic and safety characteristics of raw goat milk, whey, brine and traditional cheese during the ripening period of 28 days. The results show that the cheese under study belongs to the acid full-fat cheese group. A consumer panel attributed high scores to the goat cheese, until the 21st day of ripening. After this period, the overall features altered significantly, including bitterness, odor intensification and the development of molds on the surface. The presence of fungi, associated with Enterobacteriaceae, suggest that the hygiene of the production processes needs to be improved. The results indicate the need for better hygiene during milking and cheese production. Additionally, efforts are being made to control the production of traditionally made cheese, i.e., to place it under industrial conditions and to use pasteurized milk or starter cultures previously isolated from the same cheese in order to attain a standard of uniform quality and safety. Regarding microbial safety, the detection of putative pathogens and antibiotic resistances is recommended for an active monitoring of traditional foods to avoid foodborne infections and/or the dissemination of resistant microorganisms along the food chain.

## Figures and Tables

**Table 1:** Physico-chemical parameters of goat’s milk and whey.

		Goat Milk	Whey
Chemical characteristics	Dry matter content (%)	14.21 ± 0.13	7.7 ± 0.00
	Milk fat content according to Gerber (%)	4.60 ± 0.00	0.4 ± 0.01
	Ash content (%)	0.77 ± 0.01	0.53 ± 0.01
	Total protein content (%)	3.58 ± 0.02	1.36 ± 0.01
	pH value	6.52 ± 0.02	6.52 ± 0.02
	Titratable acidity (°SH)	6.53 ± 0.09	6.36 ± 0.04
	a_w_ value	0.945 ± 0.00	0.946 ± 0.00
Color parameters (D65)	L*	82.44 ± 0.09	39.22 ± 0.03
	a*	−3.16 ± 0.01	−2.92 ± 0.07
	b*	6.85 ± 0.00	4.53 ± 0.02
	Dominant wavelength (nm)	568.56 ± 0.02	565.17 ± 0.27

Data are presented as mean ± SD; nd—data not determined.

**Table 2:** Physico-chemical parameters of goat’s cheese during ripening.

		Day 0	Day 7	Day 14	Day 21	Day 28
Chemical characteristics	Dry matter content (%)	38.91 ± 0.16 ^a^	41.80 ± 0.11 ^b^	50.87 ± 0.28 ^c^	52.64 ± 0.13 ^d^	46.79 ± 0.06 ^e^
	Milk fat content according to Van Gulik (%)	23.00 ± 0.00 ^a^	25.83 ± 0.23 ^b^	32.25 ± 0.25 ^c^	33.5 ± 0.00 ^d^	32.75 ± 0.25 ^c,e^
	Fat in dry matter (%)	59.11 ± 0.00 ^a^	61.96 ± 0.00 ^b^	63.40 ± 0.00 ^c^	63.64 ± 0.00 ^c,d^	69.99 ± 0.00 ^e^
	Ash content (%)	2.03 ± 0.17 ^a^	1.60 ± 0.01 ^b^	1.66 ± 0.002 ^b,c^	1.66 ± 0.02 ^b,c^	1.30 ± 0.01 ^d^
	Total protein content (%)	12.31 ± 0.35 ^a^	12.54 ± 0.35 ^a^	14.34 ± 0.14 ^b^	15.31 ±0.49 ^b,c^	14.22 ± 0.36 ^b,c^
	pH value	6.55 ± 0.01 ^a^	5.30 ± 0.01 ^b^	5.15 ± 0.01 ^b,c^	4.98 ± 0.00 ^d^	4.75 ± 0.01 ^d,e^
	Titratable acidity (°SH)	8.53 ±0.37 ^a^	37.86 ± 0.99 ^b^	53.60 ± 1.60 ^c^	61.60 ± 0.80 ^d^	65.20 ±0.40 ^e^
	a_w_ value	0.941 ± 0.00 ^a^	0.937 ± 0.00 ^a^	0.931 ± 0.00 ^a^	0.929 ± 0.00 ^a^	0.939 ± 0.00 ^a^
	NaCl content (%)	0.72 ± 0.00 ^a^	0.80 ± 0.04 ^b^	0.69 ± 0.01 ^c^	0.92 ± 0.01 ^d^	0.96 ± 0.04 ^d,e^
Color parameters (D65)	L*	88.49 ± 0.69 ^a^	87.14 ± 0.00 ^a^	87.95 ± 1.19 ^a^	87.46 ± 0.77 ^a^	nd
	a*	−1.88 ± 0.04 ^a^	−1.32 ± 0.44 ^b^	−1.82 ± 0.09 ^a,c^	−1.98 ± 0.16 ^d^	nd
	b*	10.30 ± 0.43 ^a^	10.95 ± 3.21 ^a^	12.51 ± 1.11 ^b^	12.03 ± 0.37 ^b^	nd
	Dominant wavelength (nm)	573.24 ± 0.09 ^a^	574.27 ± 0.21 ^a^	573.81 ± 0.11 ^a^	573.51 ± 0.19 ^a^	nd

Data are presented as mean ± SD; nd—data not determined; means within a row marked with differen letters differ significantly (*p* < 0.05).

**Table 3:** Total number of *Enterobacteriaceae* and aerobic mesophylic bacteria in goat’s milk and cheese during ripening.

Sample	Day of Analysis	Total Number of *Enterobacteriaceae*	Total Number of Aerobic Mesophylic Bacteria
Milk ^a^	0	1.44 × 10^4^	1.63 × 10^3^
Cheese ^b^	0	9.09 × 10^4^	3.6 × 10^6^
Cheese	7	1.87 × 10^6^	5.76 × 10^10^
Cheese	14	1.24 × 10^8^	5.24 × 10^11^
Cheese	21	3.05 × 10^7^	1.20 × 10^11^
Cheese	28	1.07 × 10^5^	3 × 10^7^

^a^ CFU/mL of milk, average values of three independent experiments; ^b^ CFU/g of cheese, average values of three independent experiments.

**Table 4:** Microbiological analysis.

Species		*E. coli*		*P. mirabilis*	*R. aquatilis*	*E. cloacae*	*E. asburiae*	*Pseudomonas* spp.
Number of isolates		53	1	6	1	2	1	1
Origin (milk or cheese)		cheese	Milk	milk	milk	cheese	sheese	milk
Biochemical characteristics	Lysine	+	+	−	−	+	+	−
	Ornitine	−	−	−	−	+	+	+
	H_2_S	−	−	−	−	−	−	+
	Glucose	+	+	+	+	+	+	+
	Mannitol	+	+	+	+	+	+	−
	Xylose	+	+	+	+	+	+	+
	ONPG	+	+	+	+	+	+	−
	Indole	+	+	−	−	+	−	+
	Urease	−	−	−	−	+	+	+
	VP	−	−	+	+	−	+	−
	Citrate	−	−	+	+	+	+	−
	TDA	−	−	−	−	−	−	+
	Gelatin	−	−	−	−	−	−	−
	Malonate	−	−	−	−	+	−	−
	Inositol	−	−	−	−	−	−	−
	Sorbitol	+	+	+	+	+	−	+
	Rhamnose	+	+	+	+	+	+	+
	Sucrose	−	−	+	+	+	+	−
	Lactose	+	+	+	+	+	+	+
	Arabinose	+	+	+	+	+	+	+
	Adonitol	−	−	−	−	−	−	−
	Raffinose	−	−	+	+	+	+	−
	Salicin	−	−	+	+	+	+	−
	Arginine	+	+	−	−	+	+	+
Growth on citrate medium		Green medium	Green medium	Blue medium	Blue medium	Blue medium	Blue medium	Blue medium
Growth on HiChrome coliform agar		Blue dark/violet	Blue dark/violet	Orange/yellow	Transparent white	Light pink	Light pink	Orange/yellow
Microgen GN-A and GN-B		*E.coli*	*E. coli*	*Enterobacter amnigenus* biogroup 1	*Pantoea agglomerans*	*Kluyvera ascorbata*	*Enterobacter gergoviae*	*Klebsiella* *oxytoca*
MALDI-TOF identification		*E. coli*	*E. coli*	*P. mirabilis*	*R. aquatilis*	*E. cloacae*	*E. asburiae*	*Pseudomonas* spp.
MALDI-TOF score		2.28 to 2.52		2.40 to 2.53	2.00	2.17 to 2.37	2.33	1.80
Proteolytic activity		−	−	−	+	−	−	−
Lypolytic activity		−	−	−	−	−	−	−
Antibiotic resistance profile		GEN	GEN	AMX, TET	S	AMX, TET	S	AMX, TET

“+”—Positive reaction; “−“—Negative reaction; the antibiotic short name whenever the isolate is resistant (GEN—gentamicin; TET—tetracycline; CL—chloramphenicol, AMX—amoxicillin; S—sensitive).

## Data Availability

Data sharing not applicable.

## Supplementary Materials

The following supporting information can be downloaded at: https://www.mdpi.com/article/10.3390/microorganisms10010090/s1, Supplementary Figure S1. Plates inoculated with: A (goat milk); B (whey); C (brine); D (goat cheese) after incubation; Supplementary Figure S2. Alternaria alternata isolated from goat milk; Supplementary Figure S3. Geotrichum candidum isolated from goat milk; Supplementary Figure S4. Acremonium strictum isolated from whey; Supplementary Figure S5. Penicillium brevicompactum isolated from whey; Supplementary Figure S6. Penicillium chrysogenum isolated from whey; Supplementary Figure S7. Penicillium expansum isolated from whey; Supplementary Figure S8. Penicillium glabrum isolated from whey; Supplementary Figure S9. Talaromyces albobiverticillus isolated from whey; Supplementary Figure S10. Acremonium strictum isolated from brine; Supplementary Figure S11. Penicillium aurantiogriseum isolated from brine; Supplementary Figure S12. Penicillium glabrum isolated from brine; Supplementary Figure S13. Penicillium thomii isolated from brine; Supplementary Figure S14. Penicillium thomii isolated from brine; Supplementary Figure S15. Cladosporium macrocarpum isolated from goat cheese (0 day); Supplementary Figure S16. Penicillium aurantiogriseum isolated from goat cheese (0 day); Supplementary Figure S17. Aspergillus flavus isolated from goat cheese (21st day).
