# Supplementary material for: Serbian Traditional Goat Cheese: Physico-Chemical, Sensory, Hygienic and Safety Characteristics
Source: Microorganisms. 2021 Dec 31;10(1):90. doi: 10.3390/microorganisms10010090 (PMC8778733; doi:10.3390/microorganisms10010090)
Supplement: Supplementary file 1 [file microorganisms-10-00090-s001.zip › microorganisms-1526484-supplementary.pdf]

# Suplementary material

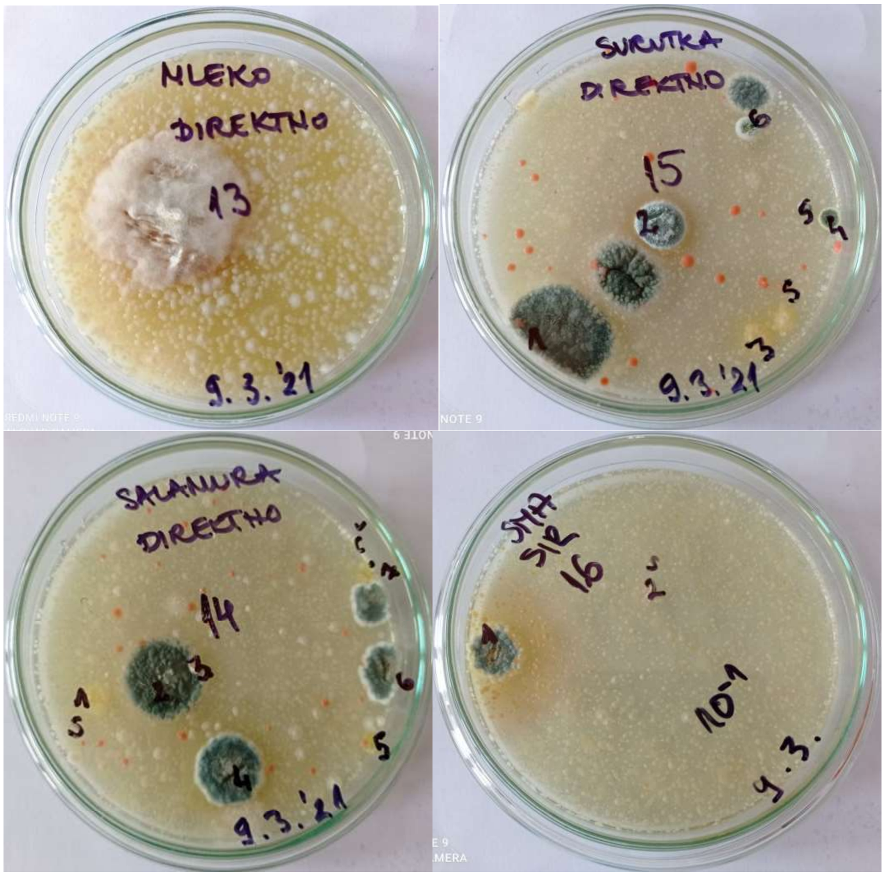


**Suplementary Figure S1.** Plates inoculated with: A (goat milk); B (whey); C (brine); D (goat cheese) after incubation

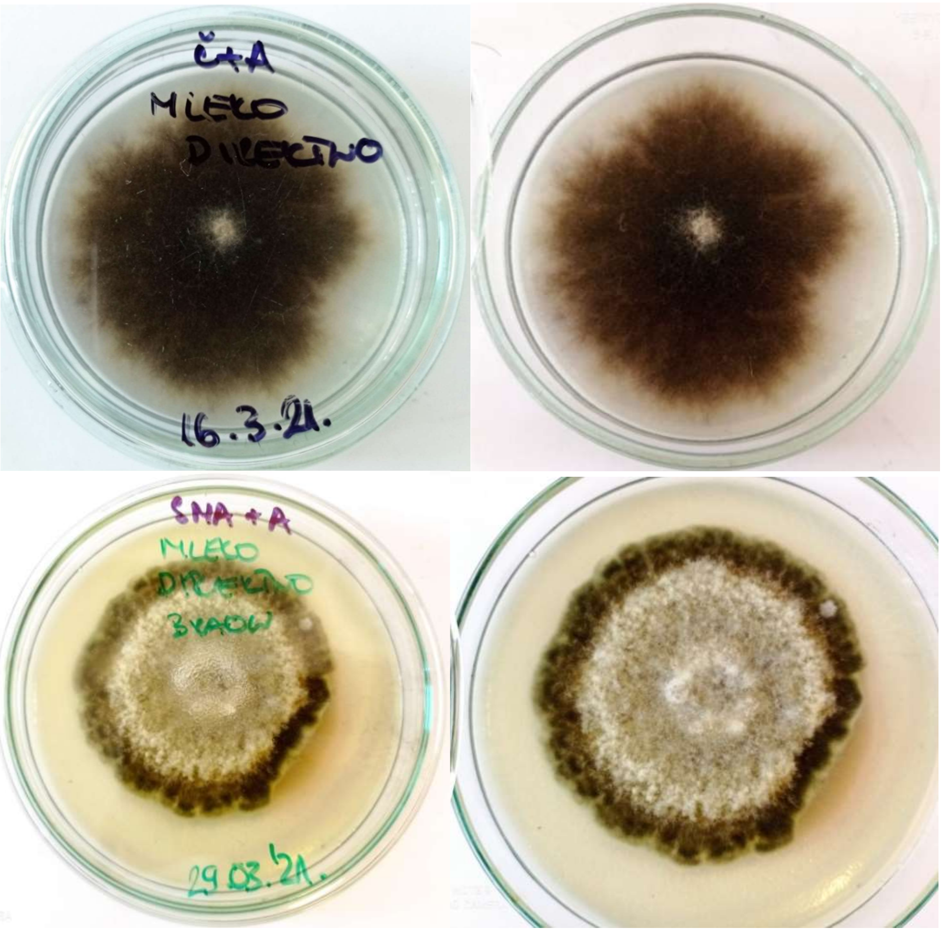


**Suplementary Figure S2**. *Alternaria alternata* isolated from goat milk

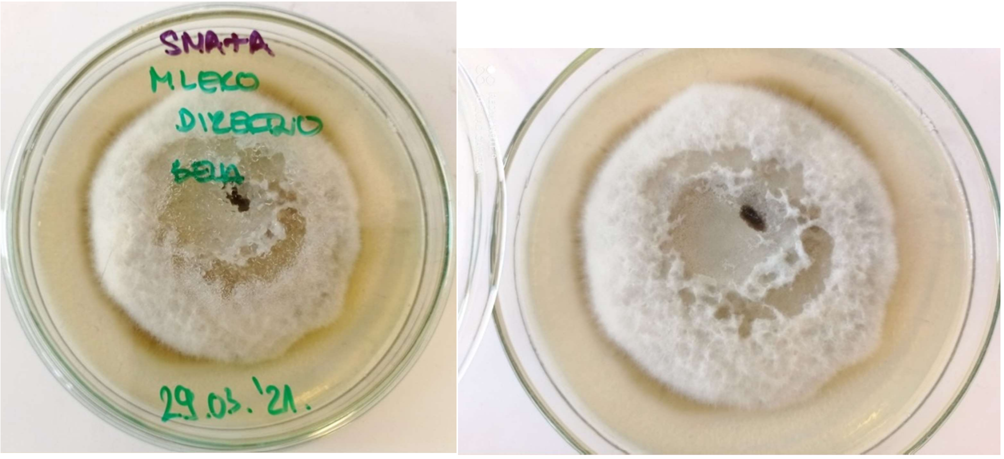


**Suplementary Figure S3**. *Geotrichum candidum* isolated from goat milk

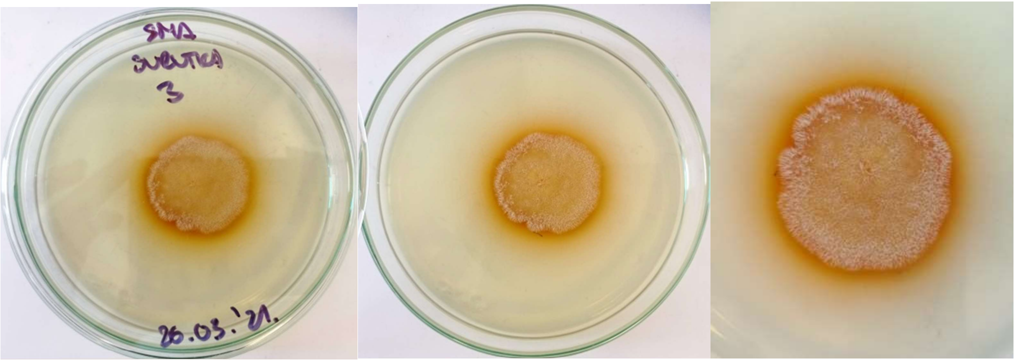


**Suplementary Figure S4**. *Acremonium strictum* isolated from whey

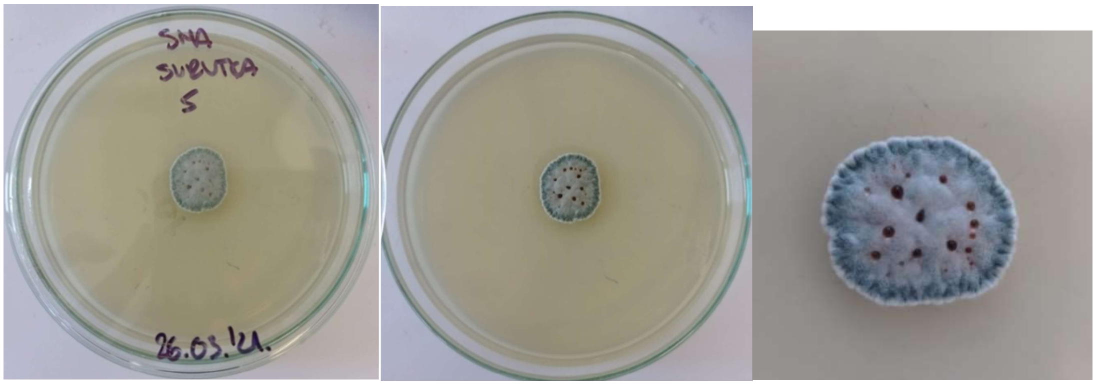


**Suplementary Figure S5**. *Penicillium brevicompactum* isolated from whey

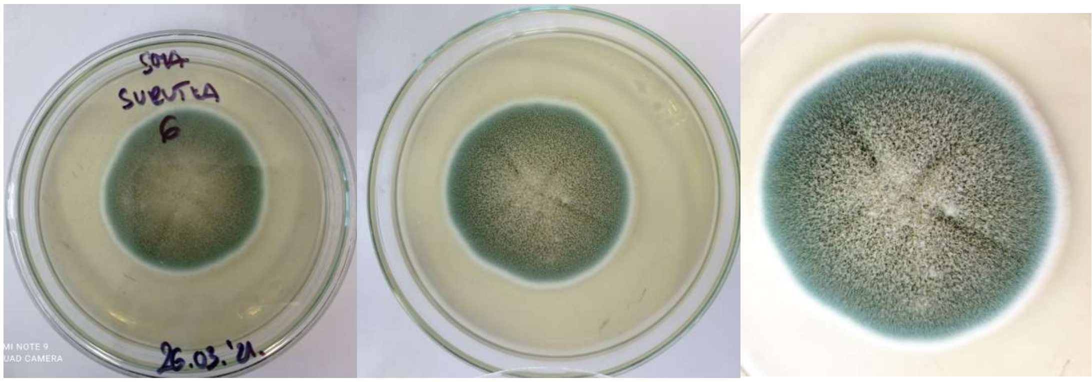


**Suplementary Figure S6**. *Penicillium chrysogenum* isolated from whey

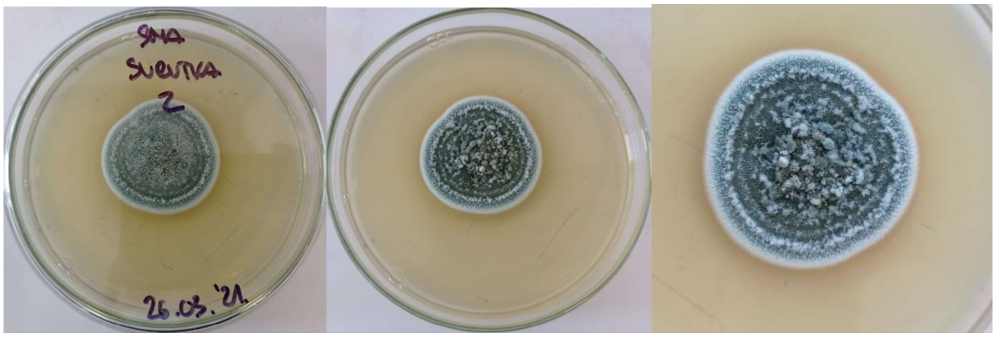


**Suplementary Figure S7**. *Penicillium expansum* isolated from whey

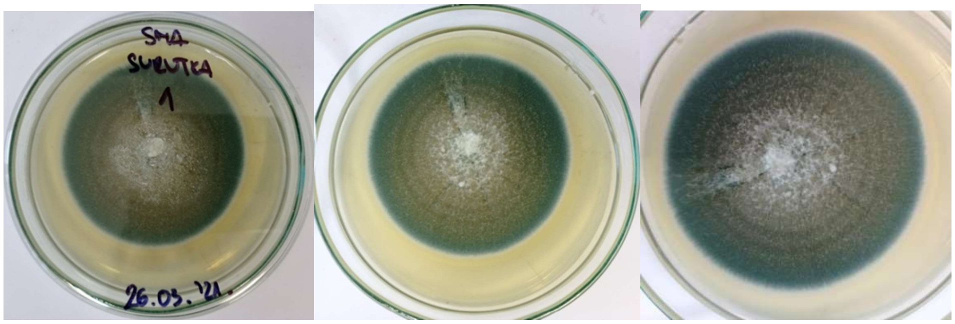


**Suplementary Figure S8**. *Penicillium glabrum* isolated from whey

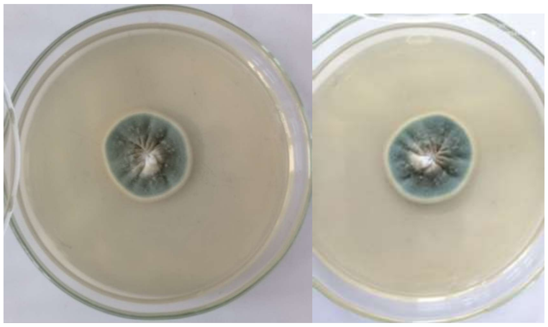

**Suplementary Figure S9**. *Talaromyces albobiverticillus* isolated from whey

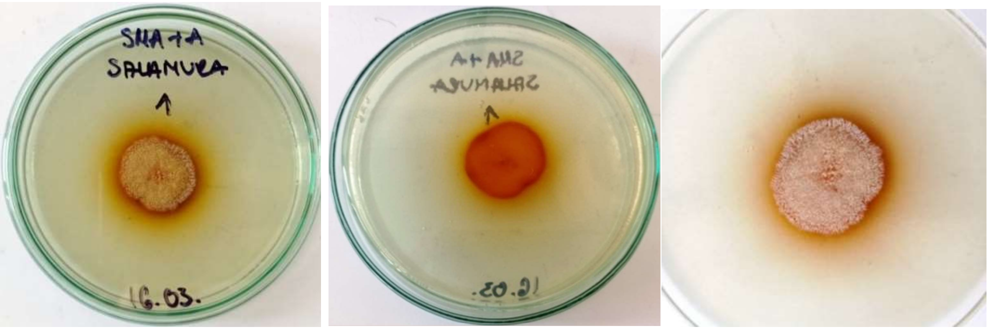


**Suplementary Figure S10.** *Acremonium strictum* isolated from brine

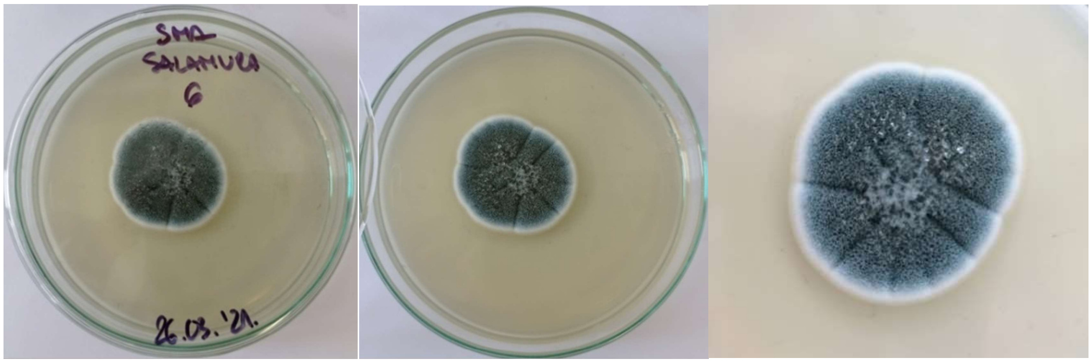


**Suplementary Figure S11.** *Penicillium aurantiogriseum* isolated from brine

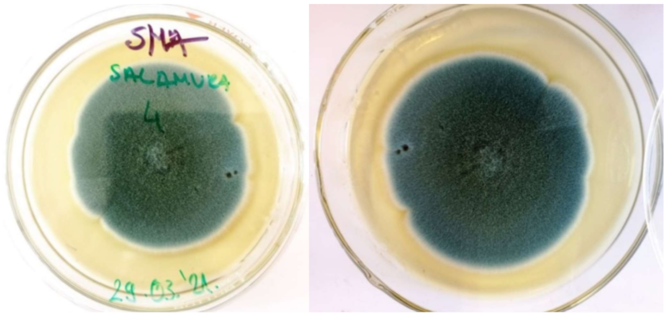


**Suplementary Figure S12.** *Penicillium glabrum* isolated from brine

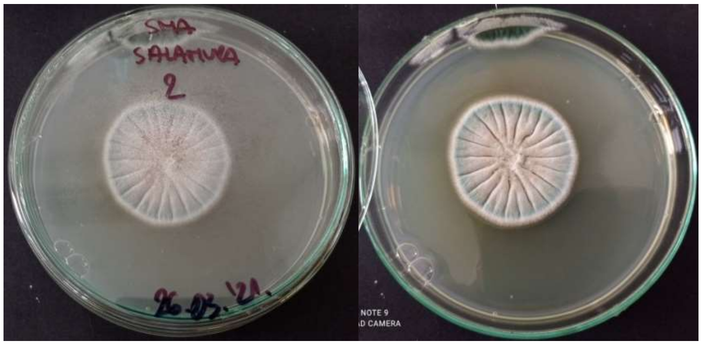


**Suplementary Figure S13.** *Penicillium thomii* isolated from brine

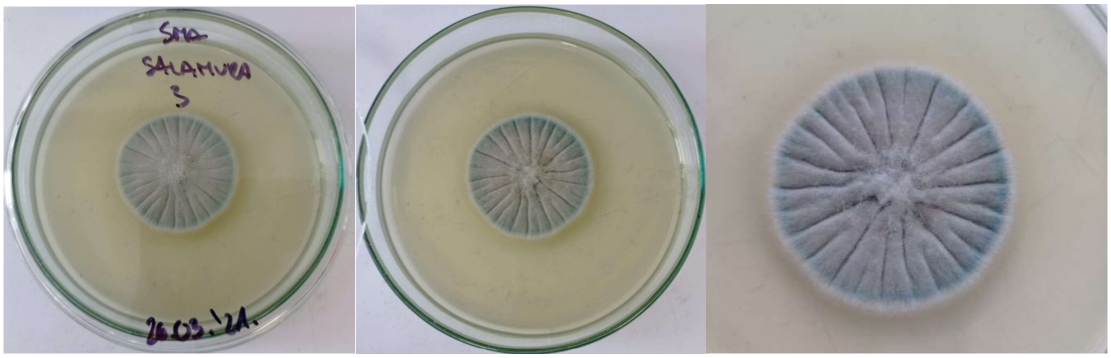


**Suplementary Figure S14.** *Penicillium thomii* isolated from brine

**Goat cheese**

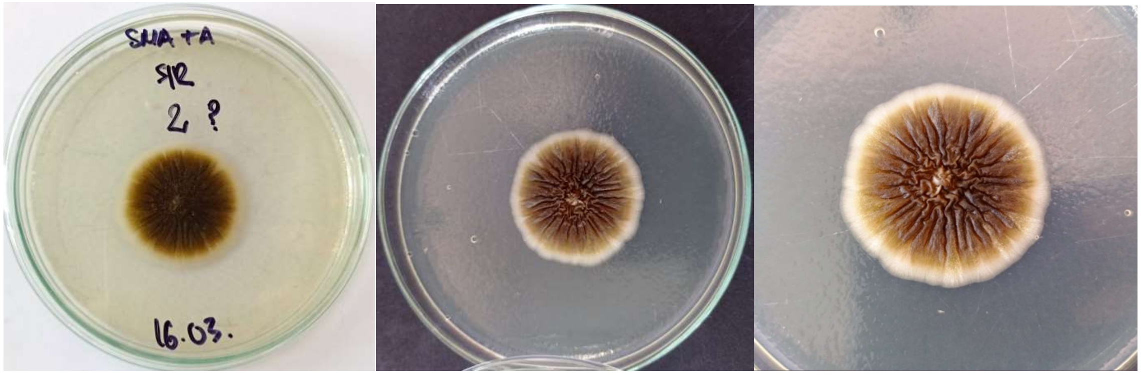


**Suplementary Figure S15.** *Cladosporium macrocarpum* isolated from goat cheese (0 day)

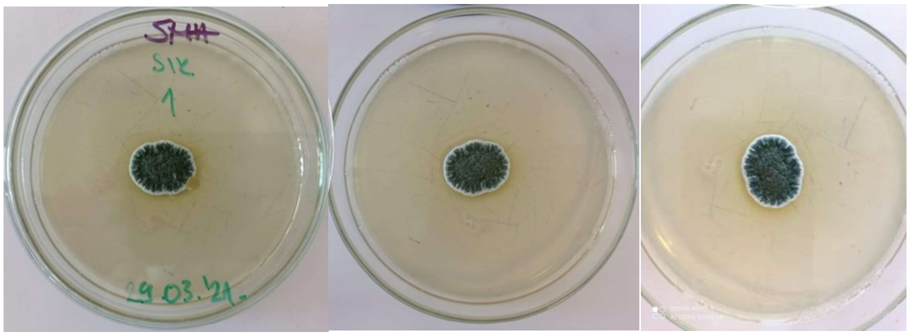


**Suplementary Figure S16.** *Penicillium aurantiogriseum* isolated from goat cheese (0 day)

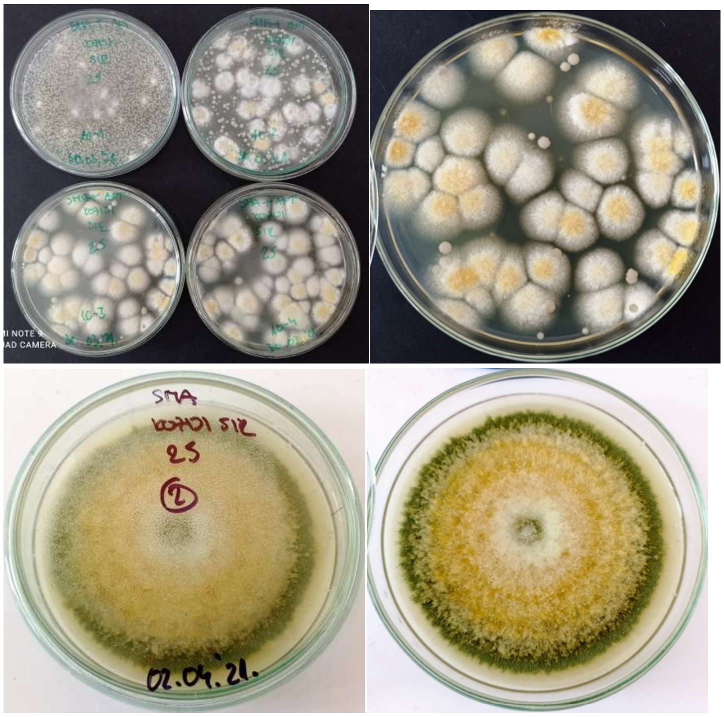


**Suplementary Figure S17.** *Aspergillus flavus* isolated from goat cheese (21st day)
